# Multiple sequence alignment accuracy and evolutionary distance estimation

**DOI:** 10.1186/1471-2105-6-278

**Published:** 2005-11-23

**Authors:** Michael S Rosenberg

**Affiliations:** 1 Center for Evolutionary Functional Genomics, The Biodesign Institute, and the School of Life Sciences, Arizona State University, Tempe, AZ 85287-4501, USA

## Abstract

**Background:**

Sequence alignment is a common tool in bioinformatics and comparative genomics. It is generally assumed that multiple sequence alignment yields better results than pair wise sequence alignment, but this assumption has rarely been tested, and never with the control provided by simulation analysis. This study used sequence simulation to examine the gain in accuracy of adding a third sequence to a pair wise alignment, particularly concentrating on how the phylogenetic position of the additional sequence relative to the first pair changes the accuracy of the initial pair's alignment as well as their estimated evolutionary distance.

**Results:**

The maximal gain in alignment accuracy was found not when the third sequence is directly intermediate between the initial two sequences, but rather when it perfectly subdivides the branch leading from the root of the tree to one of the original sequences (making it half as close to one sequence as the other). Evolutionary distance estimation in the multiple alignment framework, however, is largely unrelated to alignment accuracy and rather is dependent on the position of the third sequence; the closer the branch leading to the third sequence is to the root of the tree, the larger the estimated distance between the first two sequences.

**Conclusion:**

The bias in distance estimation appears to be a direct result of the standard greedy progressive algorithm used by many multiple alignment methods. These results have implications for choosing new taxa and genomes to sequence when resources are limited.

## Background

DNA sequence alignment is a common step in molecular evolutionary analysis. Aligned sequences are used for many purposes, including estimation of patterns of divergence, selection, the tempo and mode of evolutionary change, identification of functional elements and constraints, and phylogenetic history, just to name a few. Alignments are a hypothesis of site homology; as evolutionary distance among sequences increases, alignments are known to become less accurate [1-7]. The effect of alignment accuracy on downstream analysis in comparative genomics and bioinformatics is largely an unexplored topic, although some empirical studies have attempted to examine this with respect to functional element identification [8,9] and phylogenetic analysis [10-16].

**Figure 1:**
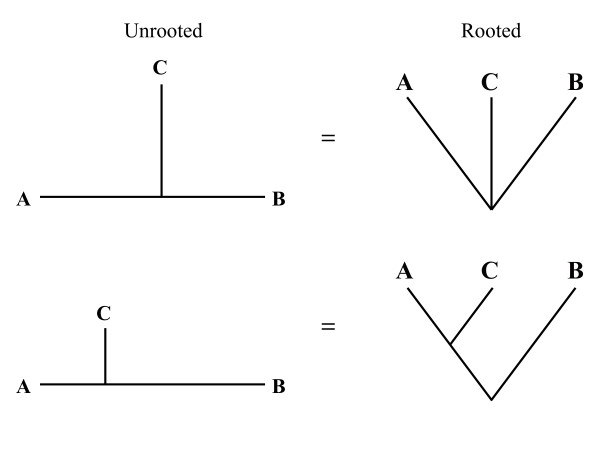
**Possible optimal locations of intermediate sequences**. Cartoon phylogenies indicating possible hypothesized optimal locations for the addition of an intermediate sequence (C) to improve the alignment of a pair of target sequences (A and B). The left column contains unrooted trees, the right column rooted trees. In the top row C is equidistant from A and B. In the bottom row, C is equidistant from A and the root of the tree.

Multiple sequence alignment, the alignment of more than two sequences, is generally thought to lead to more accurate alignments than simple pair wise alignments [4]. There are numerous approaches to multiple alignment, although most are based in some way on a progressive alignment algorithm [17,18] where similar sequences are aligned first and additional sequences are progressively added based on their divergence from the initial pair. While empirical studies have demonstrated how multiple alignments perform better than pair wise alignments [19,20], simulation methodologies have not been employed to characterize the improvement. In the simplest case, one can ask how much a pair wise alignment is improved by the addition of a third sequence (of intermediate phylogenetic position relative to the initial pair). How does varying the position of the third sequence, relative to the first two, affect the alignment? Logically, one might hypothesize that the greatest improvement will come when the third sequence is exactly equidistant from the initial pair, splitting the branch separating them in half (Figure 1); from a rooted perspective, this would be a polytomy. On the other hand, one might expect that the greatest improvement would be found from a third sequence which evenly splits a branch on the rooted tree; in an unrooted perspective this would mean the third sequence is half as close to one of the initial sequences as to the other (Figure 1).

Beyond simple accuracy, multiple sequence alignment may affect downstream sequence analysis in unexpected ways relative to pair wise sequence alignment. In a previous study [6], I show that evolutionary distance estimation from DNA sequences can be surprisingly robust to alignment error (evolutionary distance is the number of substitutions per site which have occurred since a pair of sequences diverged from a common ancestral sequence). This previous work was based on alignments of paired sequences; the relationship between accuracy of alignment and distance estimation might differ under multiple alignment conditions.

The primary goal of this study was to use simulation to examine the improvement in alignment accuracy when going from pair wise to multiple alignments, profiling the change versus the position of the additional sequences. How much is the accuracy of alignment of a pair of sequences improved by the addition of a third sequence with an intermediate evolutionary history (relative to the initial pair)? Where should the third sequence be in order to maximize the accuracy of the initial pair's alignment? In addition, the effects of these multiple sequence alignment on evolutionary distance estimation were also profiled. Does the position of the third sequence have an effect on the estimation of evolutionary distance of the initial pair, independent of the accuracy of the alignments?

## Results and discussion

### Accuracy of multiple alignments

**Figure 2:**
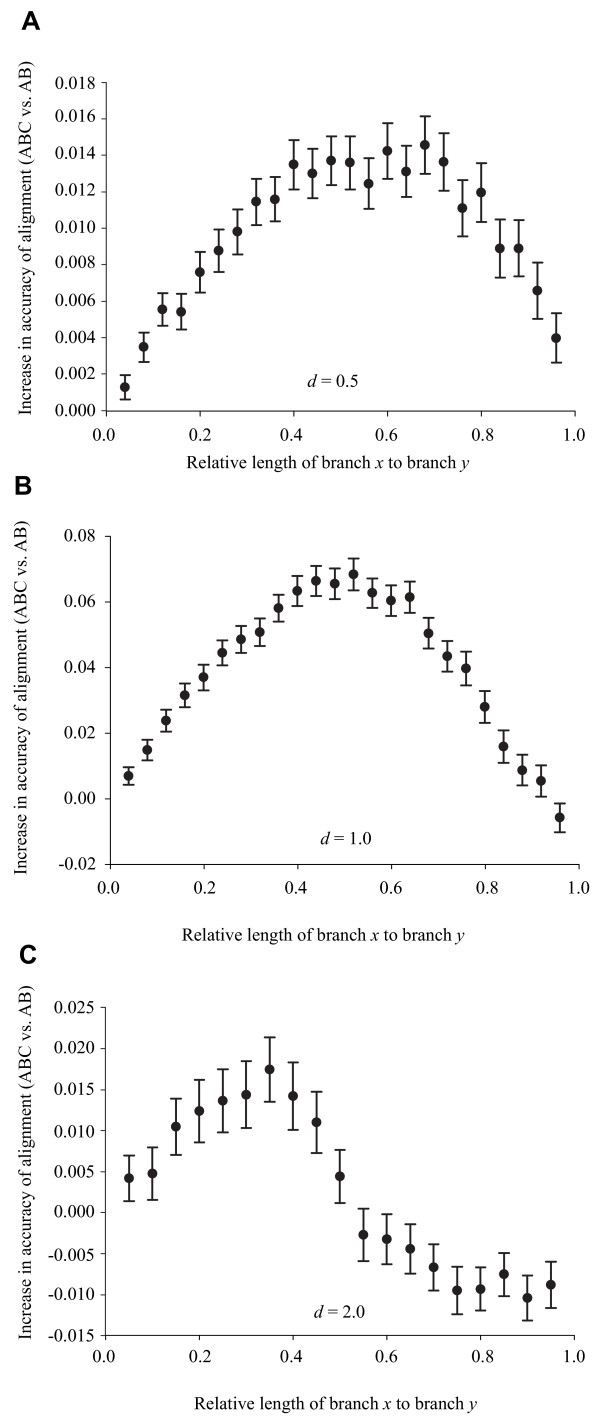
**Improvement in alignment accuracy in multiple versus pair wise alignment**. Absolute improvement in accuracy of alignment of sequences A and B in the multiple (ABC) alignment versus the pair wise (AB) alignment. Improvement was measured as ABC – AB, where ABC indicates the accuracy of the alignment of sequences A and B in the multiple alignment and AB indicates the accuracy of the alignment of these sequences in the pair wise alignment. Expected distance of sequences A and B was (**A**) *d *= 0.5; (**B**) *d *= 1.0; (**C**) *d *= 2.0. Lengths of branches *x *and *y *are illustrated in Figure 8. All points are averages of 1,000 simulation replicates. Error bars represent 95% confidence limits.

Figure 2 shows the difference in accuracy of alignment of sequences A and B between the three-sequence multiple alignments (ABC alignment) and the pair wise alignments (AB alignment) for Clustal versus the relative position of sequence C. The pattern was consistent for the two shortest divergences (Figure 2A–B): the multiple alignment was maximally more accurate than the pair wise alignment when sequence C was half as divergent from one of the target sequences than they were from each other (bottom row of Figure 1). In the final case (Figure 2C), sequences A and B were divergent to the point of being indistinguishable from random data [6]. Addition of the third sequence had a small improvement when it was relatively close to one of the original sequences, but as it moved deeper into the phylogeny it in-and-of itself became too divergent from sequence A to add any benefit to the alignment and actually marginally decreased the accuracy.

**Figure 3:**
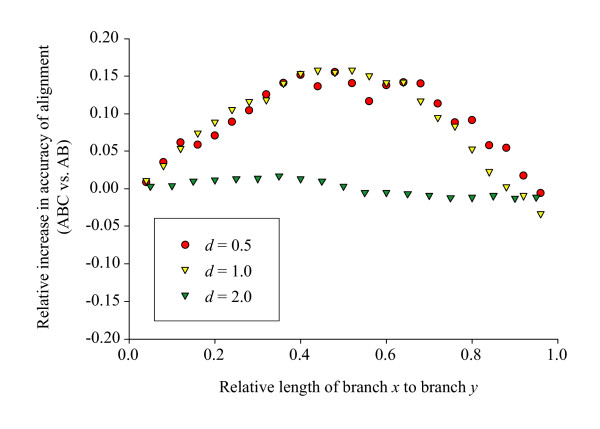
**Relative improvement in alignment accuracy in multiple versus pair wise alignment**. Relative improvement in accuracy of alignment of sequences A and B in the multiple (ABC) alignment versus the pair wise (AB) alignment. Relative improvement was measured as (ABC – AB) / AB, where ABC indicates the accuracy of the alignment of sequences A and B in the multiple alignment and AB indicates the accuracy of the alignment of these sequences in the pair wise alignment. *d *indicates the expected distance between sequences A and B. Lengths of branches *x *and *y *are illustrated in Figure 8. All points are averages of 1,000 simulation replicates.

Figure 3 shows the identical data as relative improvement in accuracy, with all three simulation conditions superimposed. The curves for the two moderate divergences (*d *= 0.5 and *d *= 1.0) were extremely congruent with both showing a peak reduction of about 15% error in the multiple alignment versus the pair wise alignment. The larger divergence (*d *= 2.0) is essentially flat since the changes in absolute error seen in Figure 2C are minimal relative to the total amount of error (~92%) in the alignments. With respect to the actual number of sites (rather than proportions), these figures correspond to a maximal increase of 12 correctly aligned sites for *d *= 0.5, 60 sites for *d *= 1.0, and 15 sites for *d *= 2.0.

**Figure 4:**
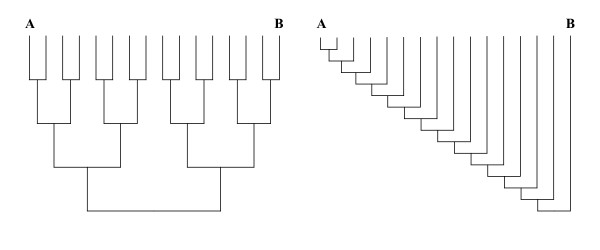
**Model trees for 16 sequence analyses**. Perfect balanced tree and perfect pectinate tree. A and B show the phylogenetic positions of these target sequences.

Additional simulations for 4-taxon trees were also performed (results not shown). As would be expected from the above results, the maximal improvement in the alignment of sequences A and B was found when the fourth sequence was added to the center of the branch leading from the root to sequence B. Simulations of 16-taxon model trees were also conducted to investigate how much improvement can be made with the addition of even more sequences. Two extreme 16-taxon trees were used as initial simulation models, a perfectly balanced tree and a perfectly pectinate tree (Figure 4). For both cases, simulations with expected AB distances of both 1.0 and 2.0 were conducted and analyzed under the same conditions as the three-taxon phylogenies (100 replicates). The expected accuracies of the AB sequence alignment from a pair wise alignment for these distances are 62% and 7%, respectively [6]. The maximal accuracies when a third sequence was added (Figure 2) were 68% and 9%. When all 16 sequences were used in a multiple alignment, the observed accuracies of the AB alignment were, respectively, 88% and 38% for the balanced tree and 77% and 19% for the pectinate tree. Unsurprisingly, adding additional sequences had a large effect on alignment accuracy when they were added in a balanced fashion and a smaller effect when they were added in a pectinate fashion. Also as would be expected, varying the internal branch lengths (including both with and without a molecular clock) or simulating across 16-taxon trees with more realistic branching patterns (e.g., non clock-like Yule trees) yielded intermediate accuracies depending on the exact shape and structure of the tree (results not shown).

These results add an additional spin to the debate over the importance of taxon sampling in phylogeny reconstruction. Although it is generally thought that increased taxon sampling yields more accurate phylogenies, simulation studies have proven to be equivocal and controversial [21-29]. However, all of these studies (and most of the empirical ones as well) tacitly assume perfect sequence alignment. Even if increased taxon sampling has no direct effect on phylogenetic accuracy (a debatable point; see cited literature above), it certain appears to have an effect on the accuracy of alignment prior to the phylogenetic reconstruction process. How alignment accuracy may directly affect phylogeny reconstruction is a topic in need of further study (although see [30]).

These results also imply a specific strategy for choosing species to sequence when resources are limited. For example, given current estimates of the mammalian phylogeny [31-33], these results suggest that error in human and mouse sequence alignments could be reduced by about 15% if aligned with a third species with an evolutionary position similar to that of the Capuchin (*Cebus albifrons*) or the blind mole rat (*Spalax judaei*). In contrast, inclusion of sequences from the completed rat genome [34], would only be expected to decrease error in human and mouse alignments by about 7%.

### Multiple alignment and evolutionary distance estimation

**Figure 5:**
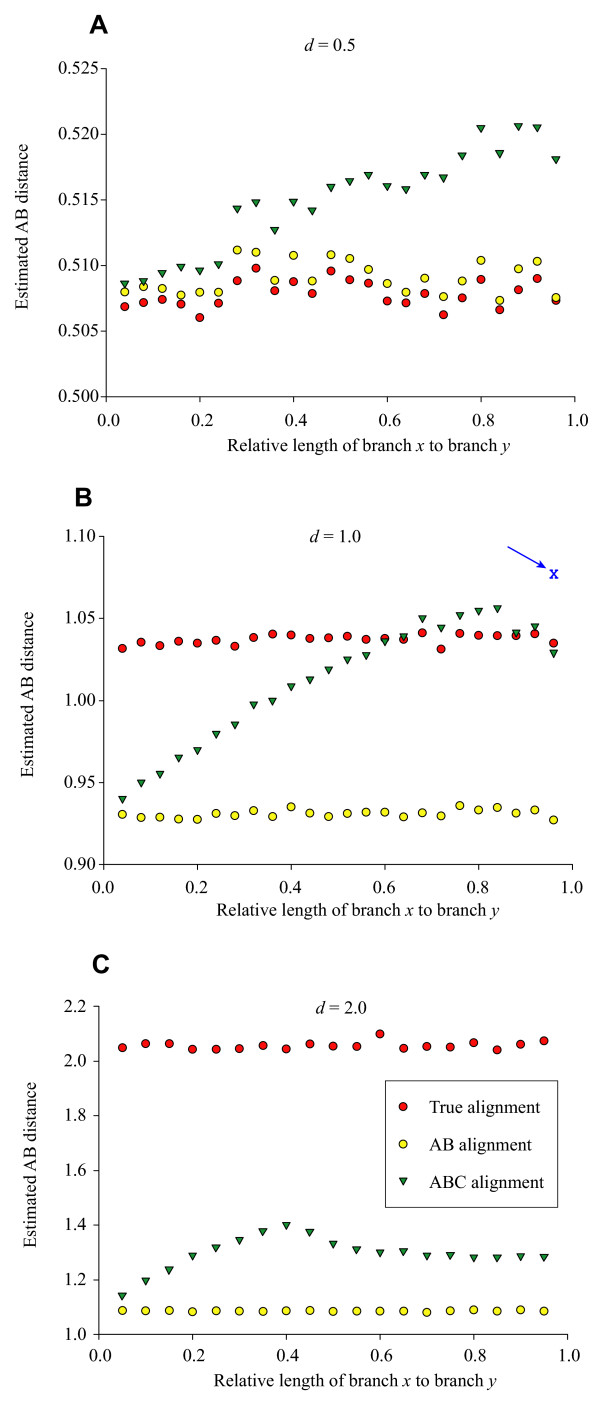
**Evolutionary distance estimates versus position of third sequence**. Estimated evolutionary distance of sequences A and B from the true, pair wise and three-taxon multiple alignments. Expected distance of sequences A and B was (**A**) *d *= 0.5; (**B**) *d *= 1.0; (**C**) *d *= 2.0. Lengths of branches *x *and *y *are illustrated in Figure 8. All points are averages of 1,000 simulation replicates. The X in panel (**B**) marked by an arrow indicates the mean distance estimate obtained for the three-taxon multiple alignments when Clustal was forced to use the correct guide tree rather than an estimating its own from the data; see the text for more information.

One would expect that estimation of evolutionary distance in a multiple alignment setting would follow that of alignment accuracy (Figures 2, 3). For alignments in Clustal, this intuition turned out to be surprisingly incorrect. Figure 5 shows the results of evolutionary distance estimation between sequences A and B from the true alignment, the pair wise AB alignment, and the multiple ABC alignments. For the intermediate distances (*d *= 0.5 and *d *= 1.0) there was a striking pattern: the estimate of distance between sequences A and B increased linearly under the multiple alignment as the branch leading to sequence C moves closer to the root. This change in distance estimation was uncorrelated with that of alignment accuracy which peaked when sequence C bisected the branch leading to sequence A (Figure 2). The slight dip at the upper end of the curves is explained by the progressive alignment procedure: pair wise alignments and distance estimates are used to set the order of sequence addition in the multiple alignment. When the branch leading to sequence C was close to the root, sequences A and B will occasionally appear to be more closely related to each other than sequence C is to A. When this occurs, A and B are aligned directly (with C on the outside) and produce the pair wise distance estimate which was consistently lower than that found from the multiple alignment (Figure 5). This was proved by realigning a subset of the data with sequence C close to the root but specifying the (correct) guide tree rather than allowing Clustal to estimate its own. The X marked by an arrow in Figure 5B shows the average distance estimate from the ABC alignments when the correct guide tree was provided; not only is the average higher than that of the original alignments but it is directly in line with the projected linear increase seen from the alignments where C branches closer to the tips. The largest simulated distance (*d *= 2.0) showed the beginning of a similar pattern to the intermediate distances (Figure 5C) before the alignments collapses into random noise (Figure 2C). The phylogenetic position of an intermediate sequence appears to bias evolutionary distance estimation, independent of alignment accuracy.

**Figure 6:**
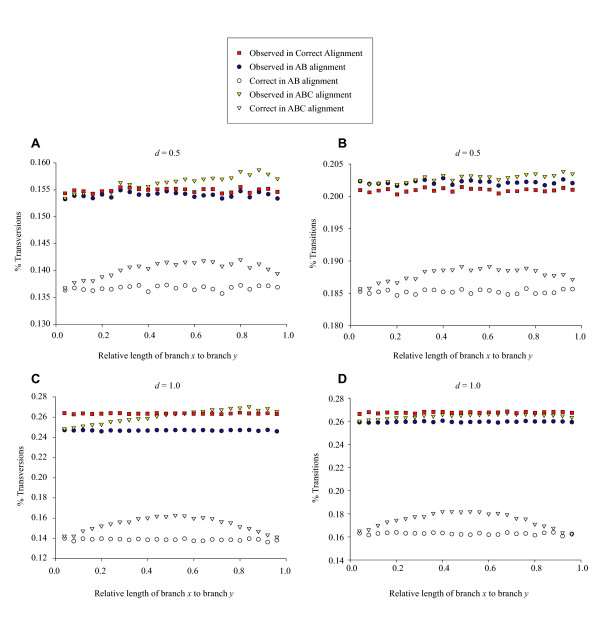
**Observed transitional and transversional sites in the alignments**. Percent of observed and correct transitional and transversional sites in the true, pair wise, and multiple sequence alignments. (**A**) Transversions for *d *= 0.5; (**B**) Transitions for *d *= 0.5; (**C**) Transversions for *d *= 1.0; (**D**) Transitions for *d *= 1.0. Lengths of branches *x *and branch *y *are illustrated in Figure 8. All points are averages of 1,000 simulation replicates.

The accuracy (or lack) of the pair wise distance estimates were exactly what one would expect from previous work [6]. To explain the observed bias in multiple alignment, I partitioned the data that goes into the calculation of evolutionary distance into its base components: observed numbers of transitional and transversional differences. Figure 6 shows the breakdown for *d *= 0.5 and *d *= 1.0. Each panel has five parts: the percent of observed sites (between sequences A and B) with the specific change (transition or transversion) in the true alignment; the percent of sites with the specific change in the AB alignment; the percent of sites with the specific change in the AB alignment that were correctly aligned (found in the true alignment); the percent of sites with the specific change found in the ABC alignment; and the percent of sites with the specific change in the ABC alignment that were correctly aligned. The true and pair wise alignment both showed the expected pattern of having no relationship between observed (or correct) percents of change with a change in the phylogenetic position of sequence C. For the multiple alignment, the proportion of sites correctly aligned (both transitions and transversions) followed the identical pattern seen in Figure 2, that is, the peak in alignment accuracy occurs when the branch leading to sequence C bisects the branch leading to sequence A. However, the observed number of transversional changes (and to a lesser extent, transitional changes) increased linearly as the branch leading to sequence C moved closer to the root (Figure 6A,C). The bias in evolutionary distance estimation in multiple sequence alignment appears to primarily be due to an overabundance of hypothesized transversional differences among the sequences.

Careful examination of the strict (greedy) progressive alignment algorithm [17,18] used by Clustal explains this pattern. When sequences A and B are aligned directly in a pair wise alignment, the hypothesized transitions and transversions are based solely on the properties of these sequences. In the multiple alignment, Clustal begins by aligning the closest pair of sequences (A and C). The more distant sequence C is from sequence A, the more transversions will have occurred since they shared a common ancestor. More transversions are therefore identified (hypothesized) between this pair during alignment. When sequence B is added to the alignment, numerous potential transversions have already been "set" between sequences A and C. Thus, any potential transversional difference between sequences A and B have less cost (on average) than would be found in the corresponding pair wise alignment because a transversion may already have been identified between sequences A and C. The biasing effect of phylogenetic position on distance estimation from pair wise alignment appears to be a consequence of the greedy progressive algorithm implemented in Clustal.

This study used Clustal because it is one of the most widely used alignment programs, particularly for high-throughput genomic analysis, and tends to be among the most accurate [7,35]. While it is quite possible that the resulting alignments could be improved by changing alignment parameters (such as the mismatch and gap costs), the purpose of this study is not to optimize the alignment but rather to examine the difference multiple alignment makes under simple conditions and to see examine downstream effects of these errors on distance estimation.

To partially examine whether the observed results are specific to Clustal or may be a more general alignment problem, most of the alignments were repeated with T-Coffee 1.37 [36]. Using the default parameters, T-Coffee produced significantly worse alignments and distance estimates than Clustal (the purpose of this study was not to compare the relative accuracy of these alignment methods and the absolute differences may be due to default parameterization choices and not the overall quality of the methods themselves). However, the shape of the alignment accuracy curves were the same (i.e., the peak gain in accuracy during multiple alignment occurred when the third sequence bisected the branch leading from the root to sequence A). The biasing effect of the position of the third sequence appeared to be less severe, and in some cases, completely absent; unfortunately the differences in accuracy made it difficult to systematically compare these results. Unlike Clustal, T-Coffee uses both global and local pair wise alignments to guide the production of the final global alignment. Although it is also a progressive algorithm, the intermediate alignments it produces make use of more information at early stages, which may help prevent the distance estimate bias.

There are many additional multiple DNA sequence alignment algorithms and programs available, some of which use similar progressive alignment schemes as Clustal and T-Coffee but allow for revision of previously aligned sequences, and some of which use very different approaches, including statistical alignments based on maximum likelihood or Bayesian methods (e.g., [37-41]). Some of these methods simultaneously estimate evolutionary distance and alignment [42,43], while methods for simultaneously estimating phylogenies and alignments are also being developed [30,44-48]. Comparisons of the overall accuracies of some of these programs in pair wise DNA sequence analysis has recently been conducted [7]. How these programs and algorithms compare under multiple alignment conditions and whether the observed biasing effect is widespread or narrow across algorithms is a task for future investigation.

## Conclusion

Multiple sequence alignments do improve upon pair wise sequence alignments. The optimal taxon sampling strategy for maximally improving alignments is to bisect long branches in a balanced framework. Independent of alignment accuracy, however, multiple alignment using a progressive algorithm can bias evolutionary distance estimates, with larger estimates consistently found as intermediate sequences appear deeper in the phylogeny.

## Methods

**Figure 7:**
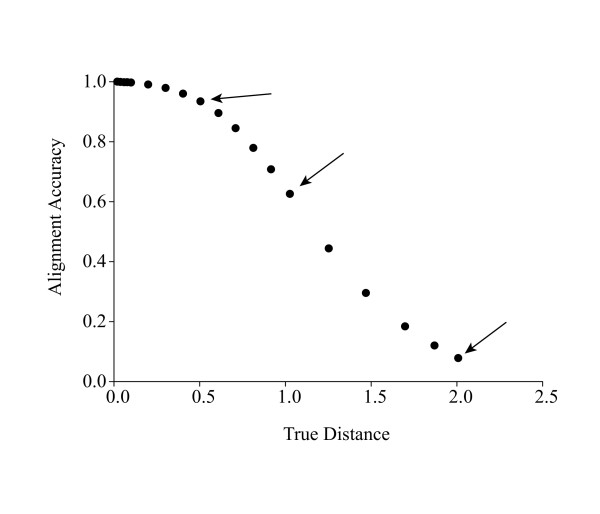
**Alignment accuracy versus true distance**. Proportion of sites correctly aligned versus true distance for the HKY substitution model. Arrows indicate the chosen divergence of sequences A and B and expected accuracy from a pair wise alignment used for the base simulations in this study. Figure modified from Rosenberg [6].

In a previous study [6], it was found that the shapes of alignment accuracy profiles (e.g., Figure 7) were largely independent of substitution model complexity, sequence length, and many model parameter choices. This study generally follows the methods from the previous study. All simulations were performed using MySSP [49]. Simulations were conducted using the Hasegawa-Kishino-Yano (HKY) model of nucleotide substitution [50]. Initial sequences consisted of 1000 random nucleotides, with initial and expected nucleotide frequencies of π_C _= π_G _= 0.3, π_T _= π_A _= 0.2. The transition-transversion bias was set to that observed at neutral sites in mammals, κ = 3.6 [51].

**Figure 8:**
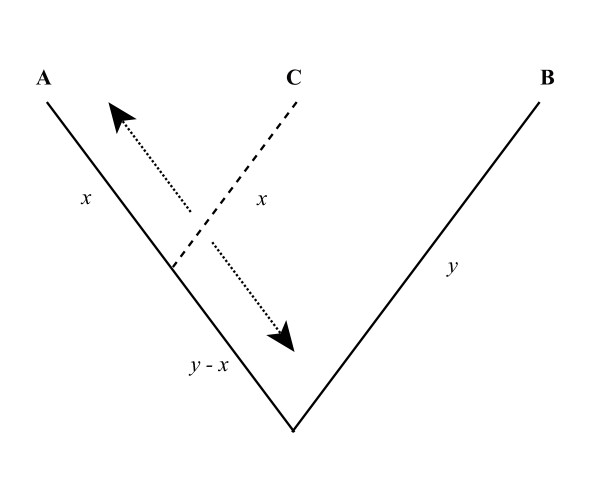
**Model tree structure**. Model tree used for the three-taxon simulations (*x *and *y *indicate branch lengths). Sequences A and B were set to a fixed divergence (*d *= 2*y*). The position of C was varied from close to A (*x *« y) to close to the root (x ≈ *y*).

Initial sequences were allowed to evolve along fixed trees representing different levels of expected divergence. The initial sets of simulation consisted of three-taxon trees (Figure 8), where the expected divergences of the target sequences (A & B) were 0.5, 1.0, or 2.0. Previous work [6] indicates that pair-wise alignments of sequences A & B at these divergences will be about 94%, 62%, and 7% accurate, respectively, under these simulation and alignment conditions (Figure 7). The location of the intermediate sequence (C) was set to a variety of evenly spaced positions ranging from close to sequence A to close to the root.

In addition to point substitutions under the HKY model, insertions and deletions were allowed to occur, with the expected rate of deletion events being one occurrence every 40 substitutions and the expected rate of insertion events being one occurrence every 100 substitutions (as observed in primates and rodents) [52]. Realized number of insertions and deletions were drawn from a Poisson distribution with mean equal to the expected value. The lengths of individual insertion and deletion events were also chosen from a truncated (so as not to include zero) Poisson distribution with a mean of 4 bases (as observed from primate and rodent lineages) [52,53]. Variation in insertion/deletion rate and size can have a large affect on alignment accuracy [6]. However, it is likely that changing the values of these parameters in the present study would have similar effects across all conditions. Each simulation condition was replicated 1000 times.

For every simulated data set, the fate of each of the original sites was tracked and an alignment representing the true homology was constructed for each data set (that is, the simulation program produced gapped sequences in which all aligned sites were truly homologous). The gaps were removed from the sequences and data sets consisting of all three sequences and of just sequences A and B were constructed. Each data set was aligned using Clustal W version 1.83 [54] with the default parameters, as is common in high-throughput analysis and comparative studies of this sort [3,6,7,36,55-57]. This produced a hypothesized alignment, just as one would obtain from analysis of real data.

The hypothesized alignments were compared to the true alignment derived from the simulation. Evolutionary distances between sequences A and B were estimated for the correct alignment, the AB hypothesized alignment, and the ABC hypothesized alignment using the Tamura-Nei formula [58].

## Authors' contributions

MR designed, programmed, executed, and analyzed all parts of this study.
